# Role of macronutrients, dairy products, fruits and vegetables in occurrence and progression of endometriosis: A summary of current evidence in a systematic review

**DOI:** 10.52054/FVVO.16.4.046

**Published:** 2024-12-27

**Authors:** N Akgun, N Sofiyeva, P.B. Yalcın, A.S. Laganà, E Oral

**Affiliations:** University of Health Science, Etlik Zubeyde Hanim Training and Research Hospital, Department of Obstetrics and Gynecology, Ankara, Türkiye; University of Bergen, Department of Clinical Science, K.G. Jebsen Center for Genome- Directed Cancer Therapy, Bergen, Norway; Private Office, Istanbul, Türkiye; Department of Obstetrics and Gynecology, “Filippo Del Ponte” Hospital, University of Insubria, Varese, Italy; Biruni University Hospital Department of Obstetrics and Gynecology, Istanbul, Türkiye; * These authors contributed equally.

**Keywords:** Endometriosis, diet, macronutrients, dairy, fruits, vegetables

## Abstract

**Background:**

Current evidence on the role of macro- and micronutrients in the aetiopathogenesis of endometriosis is controversial.

**Objectives:**

In this systematic review, we aimed to investigate the effect of macronutrients, dairy products, fruits, and vegetables on the occurrence and progression of endometriosis.

**Materials and Methods:**

A systematic literature review of eligible articles retrieved from medical databases, including PubMed, Cochrane, and Academic Search, was performed from inception to May 2023.

**Main outcome measures:**

The role of nutritional diet effects in endometriosis.

**Results:**

Our search yielded 12 studies, including five prospective cohort trials and seven case-control studies. The analysis of this literature supports the idea that processed and unprocessed red meat increases the risk of endometriosis, while no conclusive evidence exists about the effects of other protein sources on the disease. Studies on total fat consumption, including monounsaturated, polyunsaturated, saturated, and trans-unsaturated fats, do not suggest a definitive association with endometriosis. Green leafy vegetables and fresh fruit consumption may reduce the risk of endometriosis. Furthermore, the evidence regarding fibre consumption is not conclusive. Dairy products were found to have a risk-reducing effect on the disease. However, there was no consensus about the role of vitamin D in endometriosis.

**Conclusions:**

The certainty of the relationship between endometriosis and outcomes of nutritional factors was “very low” to “low,” which limits current literature from being applied for conclusive interpretations. Further large-scale randomised trials and consequent meta-analyses are recommended for high-level evidence.

**What is new?:**

This article presents an overview of evidence-based studies on the relationship between endometriosis and macronutrients. In addition, the possible influence of other nutritional variables on the development of endometriosis and the limitations of nutritional studies.

## Introduction

Endometriosis is a benign, oestrogen-dependent, chronic inflammatory disease observed in 1 in 10 women of reproductive age, primarily between 15 and 49 years (Yin et al., 2018). The aetiology of endometriosis is still under debate (Sampson, 1927; Savaris and do Amaral, 2011). Epidemiological studies investigating nutritional effects on the development of endometriosis analyse the potential impact of specific dietary components on hormone-related diseases, menstrual cycle regularity, oestrogen levels, immune and inflammatory factors, and prostaglandin metabolism (Markowska et al., 2023; Missmer et al., 2010).

The impacts of different diets on endometriosis have been vastly investigated. Studies focusing on the effects of individual dietary products indicate various directions in the disease’s pathology. Vegetarian diets, diets containing high omega-3 and low omega-6 polyunsaturated fatty acids, have risk-reducing effects (Emmett, 2017; Missmer et al., 2010). Also, fibre antioxidants and vitamin D in a plant-based diet may have a positive impact by preventing inflammation resulting in endometriosis. On the other hand, transaturatedfats, palmitic acid, red meat-rich foods, and a pro-inflammatory diet (processed foods containing sugars and saturated fats) significantly increase endometriosis risk (Demézio da Silva et al., 2021). In addition, a lower intake of pro-inflammatory foods has been shown to help in the treatment of endometriosis. Furthermore, nutritional modifications and lifestyle changes can decrease symptoms of endometriosis and increase the body’s energy levels and well- being (Barnard et al., 2023; Hu et al., 2023; Karlsson et al., 2020; Liu et al., 2023).

This systematic review explores the relationship between nutritional elements and risk for endometriosis development.

## Methods

This systematic review was developed according to the preferred reporting items for systematic reviews (PRISMA) statement (Abokhrais et al., 2018) and was registered at the PROSPERO Registration System (CRD42021276793) before starting the search.

### Literature search

Online databases, including PubMed, Cochrane, and Academic Search, were screened from inception to May 2023. The literature search was performed using the keywords: (endometriosis diet) OR (endometriosis nutrition) OR (endometriosis intake). Rayyan, an online tool for systematic reviews, was used for the screening process (Ouzzani et al., 2016).

### Selection Criteria

Studies investigating main macronutrients, which include proteins, carbohydrates, and fatty acids, were eligible for this review. PICO for the review was defined as below:

*Population*: Women diagnosed with endometriosis

*Intervention*: Dietary components, including proteins, carbohydrates, fatty acids, fruits, and dairy products.

*Comparison*: The same dietary components in women without diagnosed endometriosis.

*Outcomes*: Endometriosis occurrence and change in the severity of symptoms, such as quality of life, pain level (chronic pelvic pain, dysmenorrhea, dyspareunia etc.)

Studies reporting secondary results, published in a language other than English, unpublished data were not eligible for this systematic review.

### Screening process

Titles and abstracts of studies retrieved using the search strategy, as well as those from additional sources, were screened independently by two authors to identify studies that potentially met the aims of this systematic review. Full texts of these potentially eligible articles were retrieved and independently assessed for eligibility by two other review team members. Studies published in English were eligible for inclusion. Disagreements between authors were resolved through discussion with a third (external) collaborator. The evidence- level assessment was based on The GRADE working score group system (Group 2004).

### Data extraction

Two authors independently extracted data from articles about study features and included populations, types of intervention, and outcomes. Any discrepancies were identified and resolved through discussion (with a third external collaborator where necessary). Studies reporting dietary lifestyle factors such as caffeine, alcohol intake, herbal medicine, homoeopathy, supplementary intake of vitamins and minerals, supplement therapies, diet-induced disorders (food intolerances or allergies), and chemical- based medication were excluded. Animal and in- vitro studies also were not eligible. Due to the nature of the findings, we opted for a narrative synthesis of the results from selected articles.

## Results

**Figure 1:**
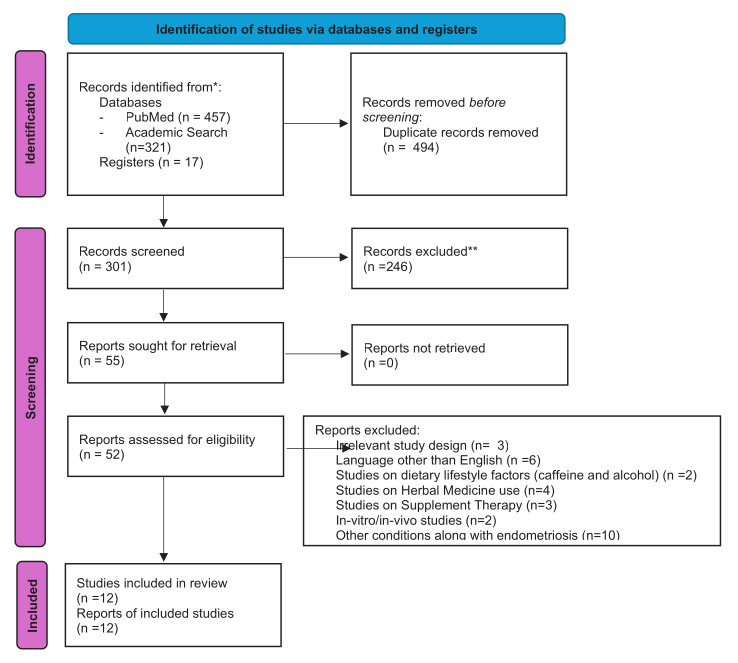
Flow Diagram and PRISMA statement.

Fifty five out of 301 articles were found relevant to the scope of this systematic review based on the title and abstract screening. Subsequent full- text screening yielded 12 studies, including five prospective cohort trials (Harris et al., 2013; Missmer et al., 2010; Nodler et al., 2020; Schwartz et al., 2022; Yamamoto et al., 2018) and seven case-control studies(Britton et al., 2000; Khanaki et al., 2012; Kim et al., 2013; Parazzini et al., 2004; Samaneh et al., 2019; Savaris and do Amaral, 2011; Trabert et al., 2011). Details of the screening process were presented in the Flow Diagram and PRISMA statement (Figure 1). The literature screening revealed previously published two meta-analyses, review articles, and an editorial opinion (Arab et al., 2022; Barnard et al., 2023; Fjerbæk and Knudsen, 2007; Halpern et al., 2015; Hansen and Knudsen, 2013; Jurkiewicz-Przondziono et al., 2017; Parazzini et al., 2013; Qi et al., 2021; Simmen and Kelley, 2018). Additionally, there were five animal studies related to the topic (Attaman et al., 2014; Covens et al., 1988; Durak et al., 2013; Heard et al., 2016; Heard-Lipsmeyer et al., 2021).

### 1. Proteins

#### 1.1. Meat

##### Red Meat

Yamamoto et al. reported that women consuming more than two servings of red meat a day (equivalent to ≥14 servings per week), mainly unprocessed red meats (hamburger, beef/pork/lamb sandwich, and liver), have a 56% higher risk of developing endometriosis than women who consume one or two times a week (Yamamoto et al., 2018). Another study which included 504 patients with laparoscopically confirmed endometriosis, demonstrated increased risk of endometriosis with a high intake of beef and other red meat (OR = 2.0) and ham (OR = 1.8) (Parazzini et al., 2004). Although most authors showed a notable equivalence between endometriosis possibility and red meat consumption, these results were opposed by a different group of investigators showing no relationship between these conditions (Trabert et al., 2011) or even the relieving effects of high animal protein intake on endometriosis signs (Samaneh et al., 2019).

##### Poultry Meat

Poultry meat proteins have many qualified and digestible proteins, unsaturated lipids (especially on the skin), low-fat content (primarily from unsaturated fatty acids), B group vitamins, and some minerals (zinc, copper, and iron) (Simmen and Kelley, 2018). Although poultry meat consumption reduces the risk of being overweight and developing obesity (Marangoni et al., 2015), 1 serving /day of poultry meat, fish, shellfish, and eggs does not affect risk of having endometriosis (Yamamoto et al., 2018).

Table I summarises the impact of meat consumption on endometriosis (Parazzini et al., 2004; Samaneh et al., 2019; Trabert et al., 2011; Yamamoto et al., 2018).

**Table I:** Review of the literature investigating the effects of fat, fish oil, and PUFA intake on the risk of the appearance of endometriosis.

Author	Year	Title	Study Design / Type of the publication	Sample size	Exposure assessment	Conclusion	Evidence level
Proteins							
Samaneh et al.	2019	The association of food consumption and nutrient intake with endometriosis risk in Iranian women: A case-control study	Case-Control Study	Iranian women (n=156) LCEP (n= 78), Healthy controls (n=78)	FFQ (147 items)	Red meat consumption is associated with a **decreased risk** of endometriosis	Very Low
Yamamoto et al.	2018	A prospective cohort study of meat and fish consumption and endometriosis risk	Prospective Cohort Study	LCEP (n= 116429)	FFQ (130 items)	More than two servings/day of red meat consumption **increases the risk** of endometriosis. The **strongest association** was found for processed red meat. **No association** was shown between the risk of poultry, fish, shellfish, and egg and endometriosis.	Low
Trabert et al.	2011	Diet and risk of endometriosis in a population-based case-control study	Case-Control Study	Women’s Health Initiative (n= 944) Endometriosis (n=284) Healthy Control (n=660)	FFQ (122 items)	Proteins are **not associated** with endometriosis risk. Animal proteins are not associated with endometriosis risk.	Low
Parazzini et al.	2004	Selected food intake and risk of endometriosis	Case-Control Study	Endometriosis (n= 504) Healthy Control (n=504)	Weekly consumption of selected dietary items	**Increased risk** with high intake of red meat (processed and or unprocessed) consumption	Low

### 2. Fatty Acids

The Nurses’ Health Study included 1199 premenopausal women diagnosed with endometriosis. This study revealed women with the highest OM-3FAs consumption are 22% less likely to be diagnosed with endometriosis than those with the lowest intake (Missmer et al., 2010). Also, Missmer et al. (2010) showed women who consumed high amounts of trans-unsaturated fats were at a higher risk of developing endometriosis than women who consumed low amounts of trans fats (RR = 1.48, 95% CI = 1.17–1.88). The results of some clinical trials suggest that women at risk for endometriosis should avoid eating foods rich in saturated fats (butter, solid margarine, lard, tail oil, whole milk and products, meat and meat products) and trans fats (French fries, muffins, cookies, biscuits, chocolate, margarine, fried chicken and crackers) (Britton et al., 2000; Samaneh et al., 2019). Britton et al. (2000) showed that polyunsaturated fatty acid (PUFAs) and vegetable fats might increase the risk of benign ovarian tumours, including endometrioid subtypes. However, some studies have emphasised that higher fat consumption is associated with a lower risk of endometriosis. Samaneh et al. (2019) found that a higher intake of total fats, PUFAs and oleic acid lowered the risk of endometriosis. In contrast, women who used butter margarine have lower endometriosis risk (Trabert et al., 2011). A third group of studies related to endometriosis has found no significant difference between the intake of fatty acids and endometriosis (Khanaki et al., 2012; Kim et al., 2013; Missmer et al., 2010; Parazzini et al., 2004; Savaris and do Amaral, 2011). Khanaki et al. (2012) demonstrated that the serum phospholipid profile was not different in endometriosis patients, but the ratio of eicosapentaenoic acid (EPA) to arachidonic acid (AA) moderately correlated with the severity of endometriosis. Also, Kim et al. (2013) long-chain omega-3 fatty acid (OM-3FA) consumption does not have a significant correlation with endometriosis. In contrast, trans unsaturated fat and a high intake of fried potatoes raise the risk of suffering from endometriosis disease (Kim et al., 2013).

Table II summarises the review of the literature evaluating the risk effects of fat, fish oil, and PUFA intake on the appearance of endometriosis (Britton et al., 2000; Khanaki et al., 2012; Kim et al., 2013; Missmer et al., 2010; Parazzini et al., 2004; Samaneh et al., 2019; Savaris and do Amaral, 2011; Trabert et al., 2011).

**Table II:** Review of the literature investigating the effects of protein intake on the risk of the appearance of endometriosis.

Author	Year	Title	Study Design / Type of the publication	Sample size	Exposure assessment	Conclusion	Evidence level
Lipids							
Samaneh et al.	2019	The association of food consumption and nutrient intake with endometriosis risk in Iranian women: A case-control study	Case-Control Study	Iranian women (n=156) LCEP (n= 78), Healthy controls (n=78)	FFQ (147 items)	**No significant difference** in intake of **total fats** **MUFAs, and PUFAs, Oleic acid, decreases** the disease risk Liquid oil and a low intake of fried potatoes reduce endometriosis	Very Low
Kim et al.	2013	Differences in omega-3 and fatty acid profiles between patients with endometriosis and those with a functional ovarian cyst	Case-Control	Korean women, n=22 Endometriosis patients, n=10	FFQ (117 items) OM-3 index n-3 PUFA n-6: n-3 ratio	Erythrocyte levels of the omega-3 index and n-3 PUFA were higher, the **n-6:n-3 ratio was lower in the endometriosis group** compared to the functional ovarian cyst group	Very Low
Khanaki et al.	2012	Evaluation of the Relationship between Endometriosis and Omega-3PUFA and Omega-6 PUFA	Case-Control	n= 120 Stage I–IV endometriosis patients, n = 46, Healthy control, n = 74 (18-42 years)	Serum phospholipid profile	Endometriosis levels and the correlation of fatty acids were **not different**	Very Low
Savaris et al.	2011	Nutrient intake, anthropometric data, and correlations with the systemic antioxidant capacity of women with pelvic endometriosis	Case-Control	n=45 LCEP, n=25, Healthy Control, n=20 (18-35 years)	24-h food recall method over 3 days Body composition assessment Antioxidant capacity analysis by total serum thiol levels measurement using DTNB (5,50-dithiobis-(2-nitrobenzoic acid)	sFA **no association** endometriosis Lower PUFAs intake in the endometriosis group compared to healthy controls	Very Low
Trabert et al.	2011	Diet and risk of endometriosis in a population-based	Case-Control	Total n= 944 Endometriosis, n=284, Control, n=660 (18–49 years)	FFQ (122 items)	Increased total fat consumption is associated with a **decreased** endometriosis risk. Women who used butter, shortening, or margarine were also associated with **decreased** endometriosis risk Saturated–monounsaturated trans-fat intake **decreased** the Endometriosis risk	Low
Missmer et al.	2010	A prospective study of dietary fat consumption and endometriosis risk	Prospective Cohort	n=70709 Endometriosis experimental group n=1199, LCEP, n=586, Healthy Control, n = 69,510 (25-42 years)	FFQ (130 items)	Total fat (Vegetable fat+ Animal fat+ Trans-unsaturated fat+ Monounsaturated fat + Polyunsaturated fat + Long-chain omega-3 fatty acids + Long-chain omega-6 fatty acids + Saturated fat) and animal fat (saturated fat and monounsatu- rated fat) consumption is **not associated** with endometriosis risk. Saturated fat, especially palmitic acid, increases endometriosis risk. 48% more diagnosed endometriosis in Trans unsaturated fat consumed group 22% less diagnosed endometriosis in PUFA Omega-3 consumed the group	Low
Parazzini et al.	2004	Selected food intake and risk of endometriosis	Case-Control Study	Endometriosis (n=1008) Healthy Control (n=504)	Weekly consumption of selected dietary items	**No association** was found between butter, margarine, and oil consumption	Low
Britton et al.	2000	Diet and benign ovarian tumors	Case-Control Study	n=1024 Benign Ovarian Tumors (n=393) Endometrioid tumors (n=280) Healthy Control (n=351)	FFQ (126 items)	Trans Fatty acids, PUFA,sFA, and MUFA **increase the risk** of being diagnosed with a subtype of endometrioid tumor.	Low

Abbreviations: LCEP - Laparoscopically Confirmed Endometriosis Patients; FFQ – Food Frequency Questionnaire; PUFA - Polyunsaturated Fatty Acid, TF – Total Fat, SFA Saturated FA, Monounsaturated Fatty Acids -MUFA

### 3. Fruits and Vegetables

The literature review reveals controversial opinions regarding the risk effects of fruits and vegetables on endometriosis (Schwartz et al., 2022). Britton et al. (2000) showed unrefined fibre intake was not associated with the risk of endometriosis; at the same time, only one study found significantly higher fibre consumption in endometriosis patients compared to the control group. A study by Harris et al. (2018) compared <2 servings per day fruit consumers with those who consumed 3, 4, 5, and ≥6 servings per day and found a 9%, 10%, 12%, and 18% decrease in endometriosis risk, respectively. The same study showed a 22% decreased risk of endometriosis in women consuming one portion of citrus fruits (orange, grapefruit, and their juices) per day (Harris et al., 2018). On the other hand, Schwartz et al. (2022) showed in a prospective cohort study that the higher the consumption of glycaemic index foods, such as vegetable fibre and cruciferous fibre, the greater the risk for endometriosis. However, women with higher intakes of fruit fibre and gluten had a lower risk of a laparoscopically confirmed diagnosis of endometriosis, although these results did not remain significant in sensitivity analyses. In addition, a case-control study reported that an increase in fruit consumption by two or more servings/day is related to higher endometriosis risk (OR 1.5, 95% CI 1.2–2.3, Ptrend = 0.04); however, this relationship was not established with vegetables (Trabert et al., 2011). Evaluation of vegetable consumption by Harris et al. (2018) did not find an association with endometriosis risk. A comparison of antioxidant intake in endometriosis and control groups showed that endometriosis patients had a lower intake of vitamins A, C, E, zinc, and copper, which have high antioxidative functions (Hughes, 2017). Other studies did not prove this fact with no relationship demonstrated between vitamin A, C, and E intake and endometriosis risk (Savaris and do Amaral, 2011; Trabert et al., 2011) .

Table III summarises review articles assessing the relationship between consuming fibre, fruits, and vegetables and endometriosis appearance risk (Britton et al., 2000; Harris et al., 2018; Parazzini et al., 2004; Samaneh et al., 2019; Savaris and do Amaral, 2011; Trabert et al., 2011).

**Table III:** Review of the literature summarizing the relationship between the consumption of fiber, fruits, vegetables.

Author	Year	Title	Study Design / Type of the publication	Sample size	Exposure assessment	Conclusion	Evidence level
Fiber, fruits, and vegetables							
Schwartz et al.	2022	Glycemic Index, Glycemic Load, Fiber, and Gluten Intake and Risk of Laparoscopically Confirmed Endometriosis in Premenopausal Women	Prospective Cohort	n=81,961 premenopausal women LCEP n=3810 to compare	FFQ (130 items)	Total vegetable and cruciferous vegetable fiber intakes were also associated with **higher risk** Higher intake of fruit fiber was associated with **lower risk of endometriosis** but the association was not significant after adjusting for the Alternative Healthy Eating Index. Gluten intake was also **associated with lower risk** **No association** was observed for Glysemic load or total, legume, or cereal fiber intake	Low
Samaneh et al.	2019	The association of food consumption and nutrient intake with endometriosis risk in Iranian women: A case-control study	Case-Control Study	Iranian women (n=156) LCEP (n= 78), Healthy controls (n=78)	FFQ (147 items)	Vegetables, especially yellow vegetables, potatoes, legumes, and fruits, are **associated with a lower risk of endometriosis**. **No association** was found between fiber consumption and endometriosis.	Very Low
Harris et al.	2018	Fruit and vegetable consumption and risk of endometriosis	Prospective Cohort	n=70 835 LCEP, n=2609, to compare Premenopausal Controls	FFQ (130 items)	There is an **inverse association** between higher fruit (especially citrus fruits) consumption and the risk of endometriosis. **No association** was found between vegetable intake and endometriosis. Only cruciferous vegetable consumption **increases disease risk**.	Low
Trabert et al.	2011	Diet and risk of endometriosis in a population-based case-control study	Case-Control	Women’s Health Initiative, n= 944 Endometriosis, n=284, Control, n=660	FFQ (122 items)	An increased number of fruit portions is related to a higher risk of endometriosis. Vegetable consumption is not associated with endometriosis.	Low
Savaris et al.	2011	Nutrient intake, anthropometric data, and correlations with the systemic antioxidant capacity of women with pelvic endometriosis	Case-Control	n=45 LCEP, n=25, Healthy Control, n=20	24-h food recall method over 3 days Body composition assessment Antioxidant capacity analysis by total serum thiol levels measurement using DTNB (5,50-dithiobis-(2-nitrobenzoic acid)	Significantly higher total daily calorie intake in the endometriosis group. Higher intake of fiber is related to **increased endometriosis risk**.	Very Low
Parazzini et al.	2004	Selected food intake and risk of endometriosis	Case-Control Study	n=1008 Endometriosis (n=504) Healthy Control (n=504)	Weekly consumption of selected dietary items	A high intake of green vegetables and fruits **reduces endometriosis risk**. Carrot consumption is not related to endometriosis	Low
Britton et al.	2000	Diet and benign ovarian tumors	Case-Control Study	n=1024 Benign Ovarian Tumors (n=393) Endometrioid tumors (n=280) Healthy Control (n=351)	FFQ (126 items)	Higher consumption of vegetables **raises the risk** of being diagnosed with a subtype of endometrioid tumor.	Low

### 4. Dairy Products

Conflicting results have been reported regarding the association between dairy intake (Harris et al., 2013; Nodler et al., 2020; Parazzini et al., 2004; Samaneh et al., 2019; Trabert et al., 2011) serum vitamin D levels (Buggio et al., 2016) and endometriosis development.

Also, some studies (Samaneh et al., 2019; Trabert et al., 2011) demonstrated an inverse relationship between endometriosis risk and dairy product consumption. A prospective 9-year follow-up study by Nodler et al. (2020) showed that women with daily consumption of more than four servings of dairy products during adolescence had a 32% reduced risk of endometriosis than women consuming one or fewer servings per day during adulthood (HR >4 servings/day, 95% CI, 0.47– 0.96; Ptrend = .04). In a prospective study, Harris et al. (2013) reported that women consuming more than three servings of dairy products per day had a lower risk of being diagnosed with endometriosis compared to ones with a consumption frequency of two servings per day (RR = 0.82, 95% CI: 0.71-0.95). It has been reported that a higher concentration of 25 (OH) vitamin D reduces the risk of endometriosis in women. These women were 24% less likely to develop endometriosis than women with the lowest vitamin D concentration (95% CI: 0.66, 0.94; Ptrend =0.003) (Harris et al., 2013). However, other studies found no relationship between vitamin D levels among endometriosis and healthy controls (Trabert et al., 2011). Also, Parazzini et al. (2004) showed no association between consuming dairye products, such as milk and cheese, and endometriosis.

A summary of studies investigating the effect of dairy products and vitamin D consumption on the risk of developing endometriosis was presented in Table IV (Harris et al., 2013; Nodler et al., 2020; Parazzini et al., 2004; Samaneh et al., 2019; Trabert et al., 2011).

**Table IV:** Review of the literature summarizing the relationship between the consumption of Dairy products and Vitamin D.

Author	Year	Title	Study Design / Type of the publication	Sample size	Exposure assessment	Conclusion	Evidence level
Nodler et al.	2020	Dairy consumption during adolescence and endometriosis risk	Prospective Cohort Study	n= 32 868 LCEP, n=581 Control, n=32 287	FFQ (124 items)	Dairy products (especially yogurt and ice cream) **reduce the risk of endometriosis** High-fat dairy nutrient, compared to low-fat dairy nutrient, is **not associated with endometriosis** risk. Vitamin D intake is not associated with endometriosis appearance.	Low
Samaneh et al.	2019	The association of food consumption and nutrient intake with endometriosis risk in Iranian women: A case-control study	Case-Control	Iranian women (n=156) LCEP (n= 78), Healthy controls (n=78)	FFQ (147 items)	Dairy products **reduce the endometriosis risk**.	Very Low
Harris et al.	2013	Dairy-food, calcium, magnesium, and vitamin D intake and endometriosis: a prospective cohort study	Prospective Cohort Study	Nurses’ Health Study 2 n = 70 556 LCEP, n=1385 Healthy Control, n=69 171	FFQ (130 items)	Total and low-fat dairy products **reduce the risk** of endometriosis. 25(OH) D **inversely associated** with endometriosis	Low
Trabert et al.	2011	Diet and risk of endometriosis in a population-based case-control study	Case-Control Study	Women’s Health Initiative, n= 944 Endometriosis, n=284, Control, n=660	FFQ (122 items)	Dairy products **decreased endometriosis risk** associated with consumption. However, this association **was not statistically significant**. 25(OH) D **is not associated** with endometriosis	Low
Parazzini et al.	2004	Selected food intake and risk of endometriosis	Case-Control Study (1984-1999)	Endometriosis (n= 504) Healthy Control (n=504)	Weekly consumption of selected dietary items	Milk and cheese consumption **is not associated** with the endometriosis.	Low

Abbreviations: LCEP - Laparoscopically confirmed endometriosis patients; FFQ - Food Frequency Questionnaire.

## Discussion

Dietary habits significantly influence metabolism through various regulatory mechanisms that lead to local and systemic hormonal changes. Food intake and appetite are also affected by sex hormones that alternate between the follicular and luteal phases of menstruation. Also, excessive consumption of high- caloric foods increases adipose tissue levels, and aromatase activity raises systemic 17-estradiol (E2) concentrations in the body (Fjerbæk and Knudsen, 2007; Grosso et al., 2017).

Several possible mechanisms could explain the relationship between meat consumption and endometriosis. In the 21st century, administering oestrogen-containing anabolic nutrients to animal foods has become a common practice to increase animal weight. Consumption of such animal products might be related to the endometriosis risk in humans (Handa et al., 2009; Huhtinen et al., 2012). Moreover, excess use of red meat may increase iron intake in the body. Excessive iron intake can increase oxidative stress, promoting chronic inflammatory processes and affecting the immune system by damaging tumour suppressor genes (Huang, 2003; Sesink et al., 2000; Ward et al., 2012).

The meta-analysis by Arab et al. (2022) showed that endometriosis disease was related to increased consumption of red meat (RR:1.17; 95%CI: 1.08- 1.26; p < 0.001; I2=82.4). The meta-analysis also found no association between poultry consumption and endometriosis risk (RR: 1.08; 95% CI: 0.98- 1.18, p = 0.104). It was also presented in some reviews that while processed or unprocessed red meat intake broadens the endometriosis disease (Fjerbæk and Knudsen, 2007), a transformation from red meat to fish or egg consumption compresses the endometriosis risk (Halpern et al., 2015; Simmen and Kelley, 2018). Also, Bernard et al. (2023) review showed that unprocessed / processed red meat and poultry meat consumption increased the endometriosis risk. However, some reviews underlined the case-control studies that some of them found no association or decreased the risk of endometriosis (Hansen and Knudsen, 2013; Parazzini et al., 2013; Samaneh et al., 2019; Trabert et al., 2011). In general, the majority of reviews and meta-analyses in studies indicate that red meat (processed/ unprocessed) is associated with increased risk of endometriosis (Arab et al., 2022; Fjerbæk and Knudsen, 2007; Halpern et al., 2015) ( Table V).

**Table V:** Summary of the literature analyzing review articles evaluating the relationship between nutrition and endometriosis appearance risk.

Author	Year	Title	Included Studies	Conclusions: Proteins	Conclusions: Fats	Conclusions: Fruits, Fiber, Vegetables	Conclusions: Dairy Products and Vitamin D
Bernard et al.	2023	Nutrition in the prevention and treatment of endometriosis: A review	Mini Review	Increased risk of endometriosis has beeen associated with red meat unprocessed/processed consumption and poultry meat	Increased risk: Palmitic acid and trans fat increased the risk No association:total fat consumption Reducing dietary fat decreases the symptoms	Increased fiber consumption decreases the endometriosis symptoms Due to antiinflamatory effects, a plant-based diet reduces symptoms	Dairy products contain estradiol and palmitic acid, which are associated with an increased risk of endometriosi Vitamin D reduces the symptoms
Arab et al.	2022	Food groups and nutrients consumption and risk of endometriosis: a systematic review and meta-analysis of observational studies	Meta-analysis 5 cohorts 3 case-control	Increased risk of endometriosis has been associated with higher red meat consumption No association Poultry meat, fish, egg	No association: TF, MUFA, PUFA İncreased the endometriosis risk SFAs,tFAs	No association: Fruits, fiber, and vegetables ( includes legumes)	A higher intake of total dairy [all low-fat and high-fat dairy foods] was associated with decreased risk of endometriosis. No associations intakes of low or high-fat dairy, cheese, or milk.
Qi et al.	2021	Relationship Between Dairy Products Intake and Risk of Endometriosis: A Systematic Review and Dose-Response Meta-Analysis	Meta-Analysis 2 cohort 5 case-control	-	-	-	Total dairy intake was inversely associated with the risk of endometriosis The risk of endometriosis decreases that dairy products ≥3 servings/day High-fat dairy and cheese intake reduced endometriosi Higher butter intake increased endometriosis No association: Whole milk, reduced fat/skim milk, yogurt
Simmen et al.	2018	Seeing red: diet and endometriosis risk	Editorial Opinion	-	-	Red meat (processed and unprocessed) raises the endometriosis risk. The substitution of red meat for poultry, fish, shellfish, or eggs decreases the risk of endometriosis.	-
Jurkiewicz-Przondziono et al.	2017	Influence of diet on the risk of developing endometriosis	Review	No association: 2 case-control studies Increased Risk: 1 case-control study	No association: 3 case-control 2 randomized-comparative studies Increased risk: 1 case-control study Decreased Risk: 3 case-control studies Risk-reducing fats: Fish oils, Omega-3 fatty acids Risk-increasing fats: trans-unsaturated fatty acids	No association: 1 case-control study Increased risk: 1 case-control study Decreased risk: 1 case-control study	No association: 2 case-control studies Decreased Risk: 1 Case-control study 1 Prospective cohort study
Halpern et al.	2015	Nutritional aspects related to endometriosis	Review of 21 articles 10 case-control 2 prospective 1 randomized-prospective 1 cohort 6 review 2 communications 2 book chapters	Red meat consumption increases the risk.	Omega 3 has an anti-nflammatory effect on endometriosis. Change in the Omega 6/ Omega 3 ratio increases menstrual pain and hormonal and autoimmune disorders in women with endometriosis. Trans fat acid increases the disease risk	Fruits, Vegetables grains exert a protective effect	Vitamin D shows a protective effect. No information was given about dairy products.
Parazzini et al.	2013	Diet and endometriosis risk: A literature review	Review 10 case-control studies 1 cohort study	Red Meat: No association: 2 Studies Increased Risk: 3 studies	Olive oil-monosaturated fat: No association: 1 study Increased risk: 1 case-control study Fish-Omega 3- PUFA: No association: 1 study Increased Risk: 1 study Decreased risk: 4 studies	Vegetable and fruit intake reduces symptoms. Increased fruit intake rises the risk 1 study Organochlorines show no association with the risk.	Dairy products do not change the risk. Vitamin D consumption decreases the risk.

Lipids are divided into two categories: saturated (remain solid at room temperature) and unsaturated (remain liquid). Fatty acids are an important source of energy and contain fat-soluble vitamins and fatty acids, which are also crucial for maintaining cell stability (Kim et al., 2013).

Olive oil is the main origin of monounsaturated fats and contains a wide range of valuable antioxidants, superoxides and other reactive species (Psaltopoulou et al., 2011). In vitro studies have shown that the endometrial fatty acid content of culture media of cells affected the survival rate of endometriotic cells; adding long-chain omega-3 fatty acids (OM-3FAs) to the culture media decreases the survival rate of endometrial cells (Grosso et al., 2017). Some reviews found that fish oil and OM3- FAs reduce the endometriosis risk (Halpern et al., 2015; Jurkiewicz-Przondziono et al., 2017). Also, Halpern et al. (2015) suggested that dietary changes in the Omega 6/ Omega 3 ratio increase menstrual pain and hormonal and autoimmune disorders in women with endometriosis. The Nurses’ Health Study II also found an inverse relationship between OM-3FA consumption and endometriosis (RR = 0.88, 95% CI = 0.62–0.99) (Shafrir et al., 2018). However, recent meta-analyses and other reviews suggest that consumption of OM-3FA has no beneficial effect on endometriosis (Arab et al., 2022; Fjerbæk and Knudsen, 2007; Hansen and Knudsen, 2013). Furthermore, Sesti et al. (2009) showed in their randomised-comparative trial that a 6-month course of hormonal suppression treatment or dietary therapy with fish oil after laparoscopic ovarian cystectomy had no significant effect on the recurrence rate of ovarian endometriosis compared to placebo. It may be due to the fact that OM- 3FAs are potential precursors of lipid mediators that impact the inflammatory process, including chemical mediators (PGE3, LTB5) (Sharma et al., 2005). Also, including small and different sample sizes affected the results.

Another topic is nutrients total fat content and trans-unsaturated fat (TFAs) effects on endometriosis. Animal studies demonstrated that high-fat diet (HFD) consumption (like the Western Diet) increases ectopic endometrial lesions and peritoneal TNF alfa levels in the immunocompetent mouse model (Heard et al., 2016). Also, the consumption of trans-unsaturated fat (TFAs) increases inflammatory markers such as IL-6 and TNF levels, which might be associated with the pathogenesis of endometriosis (Halpern et al., 2015; Hansen and Knudsen, 2013; Marcinkowska and Górnicka, 2023). Hansen and Knudsen (2013) reviewed that there was a strong relationship between trans fatty acids and OM3FA in reducing symptoms in 74,708 women. However, according to the meta-analysis, there was no relationship between total fat (RR=1.00; 95% CI: 0.93-1.08; P = 0.907), MUFA (RR=0.92; 95% CI: 0.82-1.04; P = 0.190), PUFA (RR=0.93; 95% CI: 0.86-1.02; P = 0.114) consumption and endometriosis (Arab et al., 2022). They suggest that there may be a differential association between dietary saturated fats (SFAs) and TFAs and the risk of endometriosis (TFA) (RR=1.12; 95% CI: 1.02-1.23; P = 0.019) and SFAs (RR=1.06; 95% CI: 1.04 -1.09; P < 0.001) (Arab et al., 2022). In addition, according to a review of case-control studies (Khanaki et al., 2012; Missmer et al., 2010; Parazzini et al., 2004), no clear recommendations on what relationship was observed in the majority between fats, fish oils, PUFA consumption, trans-unsaturated fat intake, and endometriosis development (Fjerbæk and Knudsen, 2007). Overall, the results are inconclusive.

Carbohydrates, which include dietary fibre (soluble and insoluble), are divided into two groups: simple carbohydrates found in ready-made food products such as sugar and complex carbohydrates and complex carbohydrates found in milk and dairy products, bread and cereals, legumes, vegetables, and fruits (Mottern, 1977).

It has been reported that high glycaemic food consumption increases levels of several cytokines in the peritoneal fluid, including interleukin (IL)-1, -6, -8, and -10, tumour necrosis factor (TNF)–α, and vascular endothelial growth factor (VEGF) (Barrier, 2010). In the study reported by Marzialli et al. (2012) 330 women with endometriosis were administered a gluten-free diet for 12 months. The authors observed a significant improvement in symptoms in 75% of the 207 women who completed the study. All women reported improved physical functioning, general health perception, vitality, social functioning, and mental health (Marziali et al., 2012). In addition, a retrospective study conducted by Moore et al. demonstrated a therapeutic benefit after four weeks of low FODMAP diet (poorly absorbed short- chain carbohydrates, including fructose, lactose, polyols, fructans, and galactooligosaccharides) administration, which reduced IBS complaints associated with deep endometriosis in 72% of patients (Moore et al., 2017). Also, Krabbenborg et al. (2021) found that eliminating gluten, dairy intake, and soya from the diet and adding vegetables reduced 70% of participants’ symptoms, resulting in a higher quality of life. Van Haaps et al. (2023) recommended that avoiding gluten, lactose, sugars, and high oestrogen nutrients (soy, sesame seeds, black beans) reduce endometriosis symptoms and improved the participants quality of life.

Epidemiological studies on nutrition mostly describe the converse association between vegetable and fruit intake and endometriosis development (Colditz et al., 1997; Fjerbæk and Knudsen, 2007; Halpern et al., 2015; Harris et al., 2018; Parazzini et al., 2004; Samaneh et al., 2019). This was explained by the possible anti-inflammatory effects of fruits and vegetables on the immune system, which plays a crucial role in lipid peroxidation activated by reactive oxygen species (ROS) and promotes cellular proliferation and angiogenesis (Soave et al., 2018). In addition, recent studies have suggested that fruits containing vitamin A precursors (alpha-beta carotene) reduce the probability of endometriosis development (Mier-Cabrera et al., 2009). Riscuta and Dumitrescu (2012) reported that green leafy vegetables, especially lettuce, and species containing important levels of folate, vitamin B6, and elements such as methionine and choline could alter gene expression through DNA methylation (Rizzo et al., 2016); thus, the consumption of green leafy vegetables is recommended (McCabe and Caudill, 2005). Mouse studies also showed beneficial effects of Vitamin C, i.e., reduction of the volume of endometrial cysts; mice receiving the highest vitamin C dose showed the lowest cyst volume (Durak et al., 2013). Also, dietary intake of high fibre through fruits and vegetables results in a significant difference in bowel movements, increases the excretion of high oestrogen levels along with faeces, and declines disease progression (Barnard et al., 2023; Kaneda et al., 1997). However, a higher intake of cruciferous vegetables, which include a lot of fibre (broccoli, cauliflower, kohlrabi, cabbage, and Brussels sprouts) regulates oestrogen metabolism in the liver and increases the oestrogen /progesterone ratio (Drummond, 2017; Kaneda et al., 1997).

Also, the meta-analysis showed that there was no association between fruit (RR 0.97; 95% CI, 0.92 to 1.02; P = 0.209) and vegetables (RR 0.97; 95%CI, 0.92 to 1.02; P = 0.256) consumption and the risk of endometriosis (Arab et al., 2022). In addition, Hansen and Knudsen (2013) reviewed 12 studies and found that vegetableand fruit intake have no association with the disease. Phytoestrogens and organochlorines may be one reason why fruits and vegetables have an adverse effect on endometriosis in some studies. The authors pointed out considerable heterogeneity in the effect sizes of the included studies (Arab et al., 2022).

Phytoestrogens are food-based oestrogens that affect the disease; the subgroups are known as isoflavonoids, commonly found in beans, soy products, and other legumes (Hughes, 2017). In a case-control study, Youseflu et al. (2020) evaluated 156 women. They showed that phytoestrogen varieties such as isoflavone, lignan, and coumestrol in dairy products and coumestrol in fruits reduce endometriosis symptoms (Youseflu et al., 2020). Mvondo et al. (2019) showed that rats fed with a diet of >10% of soy developed higher-intensity pelvic pain and an increase in the volume of ectopic loci. Rats were at an increased risk for endometriosis in adulthood if the animal was fed soy during the prepubertal stage, especially with the >10% soy content (Mvondo et al., 2019). A case-control study by Tsuchiya et al. (2007) reported an inverse association between urinary isoflavonoids and the severity of endometriosis in Japanese women.

Especially vegetables and fruits may also consist of organochlorines, which are associated with a risk of developing endometriosis (Grassi et al., 2010; Sofo et al., 2015). Organochlorines interact with the nuclear receptor in the oestrogen signalling pathway and affect endometrial cell adhesion, apoptosis, and proliferation; they may also affect the developing uterine tissue outside the uterus (Smarr et al., 2016). In a recent meta-analysis, Cano-Sancho et al. (2019) found an increased likelihood of endometriosis appearance by 1.65, 1.70, and 1.23 times with dioxins, polychlorinated biphenyls, and organochlorine pesticide exposure, respectively. However, the existing evidence is insufficient due to methodological limitations for organochlorine pesticide studies in humans; diagnostic methods required to determine asymptomatic endometriosis patients from organochlorine exposed population, difficulty in creating more accurate integrated population samples, in collecting various biological materials, including target tissues for accurate measurement of the exposure (Cano-Sancho et al., 2019).

The possible mechanisms explained by the inverse correlation between dairy product consumption and endometriosis could be related to the calcium and vitamin D content of dairy products, which regulate growth-promoting factors (insulin-like growth factor I and growth factor modulators such as negative upregulation of growth-transformed (Arab et al., 2022). In their meta-analysis, Arab et al. (2022) found that high total dairy consumption (all low-fat and high-fat dairy products) was associated with a lower risk of endometriosis (RR 0.90; 95% CI, 0.85 to 0.95; P < 0.001; I2 = 37.0%), although this association was not observed for low or high-fat dairy, cheese, or milk consumption. In addition, Qi et al.’s meta-analysis on the dose-response relationship of dairy products showed that endometriosis development reduced when the average daily intake was ≥ 3 servings (Qi et al., 2021). These inverse relationship and endometriosis risk results were supported by more recent research reporting the risk-lowering effect of dairy products on endometriosis appearance (Hansen and Knudsen, 2013). However, some reviews underlined dairy products do not change the risk of endometriosis (Fjerbæk and Knudsen, 2007; Parazzini et al., 2013). In contrast, Bernard et al. (2023) showed in their review that dairy products contain oestradiol and palmitic acid, which are associated with an increased risk of endometriosis. The different results may be the Vitamin D consumption effects, which reduce the symptoms of endometriosis (Barnard et al., 2023; Halpern et al., 2015; Parazzini et al., 2013).

Vitamin D and enzymes metabolising its receptors are found in the ovaries and endometrium of women with and without endometriosis (Buggio et al., 2016; Colonese et al., 2015) .1,25-dihydroxy vitamin D (Vitamin D3) has immune-modulating effects on cell differentiation and proliferation in normal and malignant cell types, which increase anti-inflammatory cytokines (IL-4 and transforming growth factor beta-1) and decrease pro-inflammatory cytokines (TNF-α, IL-2, and IL-6), suggesting that vitamin D may help to improve chronic inflammatory diseases (Lagana et al., 2017, Sassi et al., 2018). Furthermore, both vitamin D deficiency and endometriosis have been associated with autoimmune diseases (Nothnick, 2001). Moreover, dietary calcium intake is also inversely related to inflammatory stress (Buggio et al., 2016) and high intake may be associated with a reduced risk of endometriosis (Nodler et al., 2020).

### Limitations of Nutritional Studies in Endometriosis

Studies on diet and endometriosis did not focus on the association between other modifiable lifestyle factors such as exercise, environmental, and employment factors. Another limitation is that most investigations are observational and epidemiological studies analysing one nutrient per time. The absence of well-designed RCTs limits the ability to establish causation and provides only associative evidence of the impact of nutrition on endometriosis. Moreover, studies with short-term follow-up, including small groups without well- projected endpoints, could also be considered a limitation for better conclusions. Furthermore, the small number of studies can lead to biased conclusions or incomplete insights into the relationship between nutrition and endometriosis. Most of the studies were retrospective, with no placebo-controlled case-control studies comparing suitable matches with standardised doses, which could introduce selection and recall biases (Alom et al., 2018). This is a limitation compared with cohort studies, some of which may assess diet at multiple time points and collect data before diagnosis. In addition, our literature search revealed few original articles on this topic, and the number of reviews is higher than that of original studies.

Studies investigating the effects of dietary modifications on endometriotic lesion formation and progression are limited in the literature. The systematic review by Nirgianakis et al. (2021) showed their systematic reviews of the treatment of endometriosis with dietary interventions included three components: addition with preferred nutrients, exclusion of preferred nutrients, and total dietary alteration. We need certainly defined endpoints in trials to reduce these biases. Interpreting the findings in the context of existing knowledge, the nutritional development of the foetus influences the composition and maturation of the immune system, which in turn plays a crucial role in maintaining the metabolic and immunological activity of the gut microbiota (Indrio et al., 2022). In addition, changes in gene expression may result from dietary patterns and cell messengers, affecting epigenetic mechanisms both inside and outside the cell (Indrio et al., 2022). Excessive consumption of high-calorie foods, increased adiposity and possibly obesity may contribute to the development of endometriosis through increased aromatase enzyme activity and systemic levels of 17-estradiol (E2) (Rato et al., 2014). In addition, changes in the intracellular glucose/lipid ratio, dietary content and high levels of ROS may influence the glycaemic load and antioxidant and anti-inflammatory processes, which could lead to epigenetic changes (Farooqui and Farooqui, 2018). However, many nutritional interventions for endometriosis are reported to be successful, it is unclear whether these effects are due to the idea of controlling symptoms by following a diet or to the dietary intervention itself. It can be difficult to distinguish between the effects of diet on the risk of developing endometriosis, the effects on symptoms in patients with endometriosis, and the difference between special diets and placebo diets. Also, endometriosis- and patient-related factors that may influence the success of a dietary intervention should be clearly defined.

Furthermore, the biological mechanisms of the effects of dietary interventions should understood (Nap and de Roos, 2022) . Studies used verified, semi-quantitative, 165-item dietary data consisting of a food frequency questionnaire (FFQ) to measure nutritional habits. The disadvantage of FFQ is the inclusion of questions related to the consumption of certain foods that vary regionally and seasonally, which affects the reliability of the questionnaire. Moreover, the length and comprehensive content of the questionnaire may cause confusion, misinterpretation and unintentionally added wrong answers by participants. Therefore, it is essential to validate the FFQ according to the cultural nutrition characteristics of the studied population (Danielewicz et al., 2018). Even with a validated FFQ, it is impossible completely to exclude measurements and confounding factors that may also affect dietary behaviour, especially in terms of the variety and frequency of food consumption, BMI, energy consumption for physical activity, and resting time.

In addition, the degree of homogeneity of the study population is influenced by a number of external factors, including smoking, alcohol and drug use. Currently, one of the most important problems is exposure to a range of environmentally toxic substances such as pesticides, food packaging, food additives and polycarbonate plastic. In addition, it is clear that organochlorine pollutants in fruits and vegetables, Bisphenol A in plastic bottles, phthalates in skin care products, heavy metals and xenobiotics (antibiotics, herbicides), pharmaceutical compounds in tap water, monosodium glutamate in a variety of foods and additives to improve the taste of food contribute significantly to the impact of diet. These substances can affect molecular processes and lead to changes in nutritional parameters.

Studies show that approximately 50% of patients use nutritional changes to control the disease (Armour et al., 2019). However, one of the main problems seems to be that these nutritional plans are managed according to their experiences without scientific evidence. Also, one of the major limitations is the inconsistency of dietary intake by individuals over time (months or years) and the fact that adherence to dietary recommendations for the treatment of endometriosis can vary over time, whereby adherence can be a major challenge in the implementation of prescribed dietary regimens. In addition, nutritional attitudes to food may vary due to individual differences in eating habits, cooking skills and personal taste preferences. Diets applying complete elimination of certain foods, such as gluten-free or vegan diets, may contribute to dietary deficiencies and their effects on endometriosis is not represent in the evidence. Also, the effects of the chemicals in the foods we consume and the content of the foods may result in heterogeneity between studies. In-vitro cell culture studies depending on diet models and prospective randomised controlled trials are required to define the molecular basis of the effect on endometriosis. In terms of future work, using artificial intelligence systems to best compare nutrient factors by grouping them according to macronutrient components would be interesting. In the future, for accurate analysis of image and quantity diagnostics and nutrient interactions, personalised nutrition problems can be solved through the application of artificial intelligence (AI).

## Conclusions

In summary, several studies investigated the effect of different diets on endometriosis. Although there are contradictory results for each nutrient in the literature and high-level studies are required, most evidence supports that red meat, trans fat oils, refined sugar plus derivatives and poor antioxidants can increase the risk of endometriosis, while other studies argue the opposite. In addition` results have shown that diets rich in fresh fruits, green leafy vegetables, omega-3 unsaturated fats, and dairy products decrease the risk of endometriosis development. More significant efforts will increase the level of evidence and be revolutionary in planning the most beneficial personalised treatment for the patient.
